# Acute right-sided transcutaneous vagus nerve stimulation improves cardio-vagal baroreflex gain in patients with chronic heart failure

**DOI:** 10.1007/s10286-024-01074-9

**Published:** 2024-10-14

**Authors:** Francesco Gentile, Alberto Giannoni, Alessandro Navari, Eleonora Degl’Innocenti, Michele Emdin, Claudio Passino

**Affiliations:** 1 Health Science Interdisciplinary Center, Scuola Superiore Sant’Anna, Piazza Martiri della Libertà 33 (56127), Pisa, Italy; 2 Fondazione Toscana Gabriele Monasterio, Pisa, Italy

**Keywords:** Transcutaneous vagus nerve stimulation, tVNS, Tragus, Baroreflex, Heart rate variability, Neuromodulation, Heart failure

## Abstract

**Purpose:**

The aim of this paper is to investigate the acute effects of short-term transcutaneous vagus nerve stimulation (tVNS) on cardio-vagal baroreflex gain and heart rate variability in patients with chronic heart failure (CHF).

**Methods:**

A total of 16 adults with CHF and left ventricular ejection fraction (LVEF) < 50% in sinus rhythm were enrolled (65 ± 8 years, 63% men, LVEF 40 ± 5%, 88% on beta-blockers, 50% on quadruple CHF therapy). Over a single experimental session, after a 10-min baseline recording, each patient underwent two trials of 10-min tVNS (Parasym Device, 200 µs, 30 Hz, 1 mA below discomfort threshold) at either the right or left tragus in a randomized order, separated by a 10-min recovery.

**Results:**

Compared with baseline, tVNS did not affect heart rate, blood pressure, and respiratory rate (*p* > 0.05), and no patients complained of discomfort or any adverse effect. Right-sided tVNS was associated with a significant increase in cardio-vagal baroreflex gain (from 5.6 ± 3.1 to 7.5 ± 3.8 ms/mmHg, ∆ 1.9 ± 1.6 ms/mmHg, *p* < 0.001), while no change was observed with left-sided tVNS (∆ 0.5 ± 2.0 ms/mmHg, *p* = 0.914). These findings were independent of stimulation-side order (excluding any carry-over effect) and consistent across sex, LVEF category, and HF etiology subgroups (*p*-value for interaction > 0.05).

**Conclusions:**

Acute right-sided tVNS increases cardio-vagal baroreflex gain in patients with CHF and LVEF < 50%, with no tolerability concerns.

**Supplementary Information:**

The online version contains supplementary material available at 10.1007/s10286-024-01074-9.

## Introduction

Autonomic imbalance, characterized by increased sympathetic activity and vagal withdrawal, is a pathophysiological determinant of chronic heart failure (CHF) [1]. Although direct evidence linking increased sympathetic nerve activity, assessed through microneurography, to morbidity and mortality in CHF patients is limited [2], substantial indirect evidence supports the critical role of sympathovagal imbalance [3–6], sustained by the resetting of visceral feedbacks (namely baroreflex, chemoreflex, and ergoreflex) [7–11], in these patients.

While the vascular–sympathetic limb of the baroreflex is the main determinant of blood pressure regulation, reduced cardio-vagal baroreflex gain has demonstrated strong prognostic significance in patients with cardiovascular disease. Most notably, in the ATRAMI study, which enrolled 1284 patients with recent myocardial infarction, a lower cardio-vagal baroreflex gain was associated with a threefold higher risk of cardiac death during follow-up, independent of confounders [7]. These findings have been confirmed in patients with CHF and were not affected by the use of beta-blockers [8] or other anti-neurohormonal drugs [9]. Although the precise mechanisms remain unknown, the evaluation of cardio-vagal baroreflex gain has been proposed as a surrogate of the parasympathetic cardiovascular control and, therefore, of the overall autonomic balance. Indeed, although reduced cardio-vagal baroreflex gain was associated with other markers of disease severity [8, 9], clinical variables explained only 43% of its variance in patients with CHF [8]. Accordingly, the use of baroreceptor activation therapy (BAT) has been tested in CHF patients, showing benefits on quality of life, exercise capacity, and levels of neurohormonal activation [12]. Nevertheless, data on hard outcomes are awaited, while the invasive nature of BAT remains a major drawback. [13]

The low-level electrical stimulation of the afferent fibers of the auricular branch of the vagus nerve [i.e., transcutaneous vagus nerve stimulation (tVNS)] is emerging as a noninvasive alternative to improve sympathovagal balance [14]. On the basis of encouraging preclinical studies [15–17], tVNS has been shown to reduce sympathetic activity and increase heart rate variability (HRV) in healthy individuals [18, 19], and to improve quality of life, and inflammatory markers in patients with CHF with preserved left ventricular ejection fraction [HFpEF; left ventricular ejection fraction (LVEF) ≥ 50%] [20]. Though the precise mechanisms are still unclear, some evidence from healthy individuals suggests that the beneficial effects of tVNS may be associated with an upward resetting of baroreflex operating point [19, 21]. Nevertheless, the autonomic effects of tVNS have never been tested in patients with CHF with mildly reduced LVEF (HFmrEF; LVEF 41–49%) and patients with CHF with reduced LVEF (HFrEF; LVEF ≤ 40%), who could benefit from this neuromodulation strategy. [22]

The optimal stimulation side (i.e., right versus left ear) is however unclear [23]. On the basis of the preclinical evidence that the stimulation of the right vagus may result in a stronger effect on heart rate [24, 25], the left side has been arbitrarily chosen in most clinical studies testing tVNS to minimize bradycardia, even if in the absence of well-designed comparative studies. [23]

Therefore, this study aimed to assess the effects of right-sided versus left-sided tVNS on cardio-vagal baroreflex gain in patients with CHF and LVEF < 50% on guideline-recommended therapies.

## Methods

### Subjects

The research protocol has been approved by the locally appointed ethics committee, according to the Declaration of Helsinki and privacy rules. The presented findings are derived from the pilot phase of the Transcutaneous Vagus Nerve Stimulation in Patients with Chronic Heart Failure (TRAGUS-HF; NCT06355388) study, which will explore the autonomic, biohumoral, and functional consequences of acute and chronic tVNS in patients with HFmrEF and HFrEF.

Consecutive stable adults with a diagnosis of CHF and LVEF < 50% according to the latest guidelines were screened to be enrolled in the study [26]. Only patients in sinus rhythm were selected, and patients with implanted cardiac devices [namely, pacemakers, implantable cardioverter-defibrillators, cardiac resynchronization therapy, BAT, invasive vagus nerve stimulation (VNS), and cardiac contractility modulation] were excluded. Other exclusion criteria were any condition of clinical instability including acute coronary syndrome, acute decompensated heart failure, or therapy changes within 3 months; severe renal, hepatological, and pulmonary diseases; and neurological conditions associated with dysautonomia (e.g., diabetic neuropathy and Parkinson’s disease).

All patients underwent a comprehensive cardiological evaluation, including clinical assessment [i.e., anthropometric measures, comorbidities, New York Heart Association (NYHA) functional class, and therapies] and resting transthoracic echocardiography (Philips iE33 or EPIQ7, Andover, Massachusetts, USA) to assess LVEF. [27]

### Study procedures

Following the clinical assessment and a first familiarization with the laboratory environment and instrumentation, each patient underwent the study session on a different day, in the same quiet environmental conditions and time window (between 11 a.m. and 4 p.m.). All patients were asked not to consume coffee, tea, or alcohol in the 12 h before the study, to avoid large meals and physical exercise in the 6 h before the study, and to empty their bladder before the protocol. The intake of prescribed drugs was not changed.

All recordings were performed with the experimental subject lying semi-recumbent on a chair with the back at 45° and were asked to not speak, to breathe normally, and to stay awake. The following signals were then acquired: surface echocardiogram (ECG) through three chest electrodes (BioAmp, PowerLab, ADInstruments, Sydney, Australia), sampled at 2 kHz (bandpass 0.3 Hz–1 kHz); beat-to-beat finger blood pressure (BP) by pulse plethysmography (Finapres Medical Systems BV, Enschede, Netherlands), sampled at 400 Hz and calibrated with an arm sphygmomanometer; and respiratory rate through a belt transducer (ADInstruments), sampled at 100 Hz. All signals were stored on a computer via a data acquisition and analysis system (PowerLab 16SP™ and LabChart™, v7.1.2.5 software; ADInstruments).

### Study protocol and tVNS

For each patient, the protocol lasted consecutive 40 min. The first 10 min consisted of signals recording without tVNS, i.e., “baseline.” Left-sided or right-sided tVNS (as detailed below) was then applied. The order allocation (left-sided tVNS versus right-sided tVNS first) was randomly selected (1:1) for each patient, and the two tVNS phases were separated by a 10-min recovery. The Parasym Device (Parasym Ltd, London, UK) was used to deliver tVNS at the tragus level, as previously reported [20]. The electrical current was continuously applied for each phase with the following parameters: pulse amplitude 200 µs, frequency 30 Hz, and intensity 1 mA below the discomfort threshold (as assessed for each patient and stimulation side 30 min before starting the protocol).

### Data analysis

All the recorded signals were analyzed by one of the investigators (E.D.E.), blinded to the allocation of experimental phases (i.e., baseline, left/right-sided tVNS, and recovery).

For each phase, the signals recorded in the last 5 min were averaged and analyzed. Respiratory and BP signals were analyzed through LabChart™. Heartbeat series were extracted by using the HRV tool of LabChart™ and analyzed through Kubios (Kubios HRV 2.2, Kuopio, Finland). The raw normal-to-normal (NN) interval tachograms were visually inspected to assess the quality of signal acquisition, and artifacts were automatically corrected using a piecewise cubic spline interpolation method. A first-order detrending method was applied to remove low-frequency aperiodic trends. From each series, heart rate variability (HRV) was assessed according to the Task Force rules [28]. For time-domain HRV, the standard deviation of the NN intervals (SDNN) and the root mean square of the successive differences between NN intervals (rMSSD) were calculated [28]. For frequency-domain HRV, a power spectrum analysis of NN intervals using a fast Fourier transform was performed, applying a Welch’s periodogram (256 s window with 50% overlap) to reduce spectral leakage. The areas under the low frequency (LF; 0.04–0.15 Hz) and high-frequency (HF; 0.15–0.40 Hz) band were calculated and expressed as power (HF_power_ and LF_power_, ms^2^), Ln-transformed power, normalized units [HF_nu_ = HF/(HF + LF) and LF_nu_ = LF/(HF + LF), n.u.], and LF/HF ratio [28].

Cardio-vagal baroreflex gain was calculated through the standard deviation (SD) method, as the ratio between SDNN and the corresponding SD in systolic BP (SDsBP) [29].

$$Cardio - vagal \, baroreflex \, gain \, \left( {ms/mmHg} \right) = SDNN\left( {ms} \right)/SDsBP\left( {mmHg} \right)$$

Briefly, this method is based on the ratio between the global rather than specific variabilities of the successive NN intervals and sBP [29]. Validated against other six established methods for spontaneous cardio-vagal baroreflex gain assessment [29], the standard deviation method showed prognostic significance in a large cohort of CHF patients [9]. As originally proposed, cardio-vagal baroreflex gain was calculated for 5-min series, after linear detrending and interpolation of ectopic beats. [29]

### Statistical analysis

Statistical analysis was performed by using SPSS (version 25.0, 2017, IBM Statistics, Armonk, New York, USA), and R software (version 3.4.0), and a two-tailed *p*-value ≤ 0.05 was considered significant. Quantitative values were tested for normal or skewed distribution (Kolmogorov–Smirnov test) and reported as mean ± standard deviation (SD), or median (interquartile range), as appropriate, and qualitative values as numbers or percentages.

Baseline data were reported and compared between HFmrEF versus HFrEF patients.

As anticipated, the values of vital parameters, cardio-vagal baroreflex gain, and time- and frequency-domain HRV averaged in the last 5 min of baseline recording, left-sided, and right-sided tVNS were averaged and compared. The baseline was considered the reference category. A one-way analysis of variance (ANOVA) test for repeated measures was used to compare variables across the protocol phases. In the case of statistical significance, pairwise comparison were performed using a Bonferroni post hoc test. Subgroup analyses were performed to assess the possible influence of simulation-side order allocation (left-first versus right-first), patient sex (men versus women), LVEF class (HFmrEF versus HFrEF), and CHF etiology (ischemic versus nonischemic) on the effects of tVNS on cardio-vagal baroreflex gain.

## Results

### Patient population

Out of 18 patients selected, 2 were excluded since they were unable to maintain the semi-recumbent position for the whole duration of the study protocol, due to orthopedic reasons. Finally, 16 patients with either HFmrEF or HFrEF (8 per group) were included in the study (Table 1). The mean age was 65 ± 8 years, most patients (63%) were men, and half had an ischemic etiology of CHF, with a mean LVEF of 40 ± 5%. Patients were mildly symptomatic (NYHA class II in 63% of the cases), and received an optimal medical therapy as recommended by the latest guidelines. Of note, 88% of the patients were on beta-blockers, titrated up to 38% (25–94%) of the recommended target dose.

**Table 1:** Characteristics of the study population distinguished into HFmrEF and HFrEF categories

Variables	All patients (*n* = 16)	HFmrEF (*n* = 8)	HFrEF (*n* = 8)	*p*-Value
Clinical features				
Age, years	65 ± 8	66 ± 8	64 ± 9	0.620
Men, *n* (%)	10 (63)	6 (75)	4 (50)	0.608
BMI, kg/m^2^	27 ± 6	30 ± 5	23 ± 5	0.013
Ischemic etiology, *n* (%)	8 (50)	4 (50)	4 (50)	1.000
Hypertension, *n* (%)	8 (50)	6 (75)	2 (25)	0.132
Diabetes, *n* (%)	4 (25)	2 (5)	2 (25)	1.000
COPD, *n* (%)	2 (13)	0 (0)	2 (25)	0.467
NYHA class I, *n* (%)	6 (37)	3 (37)	3 (37)	1.000
NYHA class II, *n* (%)	10 (63)	5 (63)	5 (63)	1.000
LVEF, percentage (%)	40 ± 5	44 ± 3	36 ± 3	< 0.001
Treatments				
Beta-blockers, *n* (%)	14 (88)	8 (100)	6 (75)	1.000
Beta-blockers, dose percentage (%)	38 (25–95)	38 (24–94)	44 (26–91)	0.970
ACEi/ARB, *n* (%)	4 (25)	2 (25)	2 (25)	1.000
ARNI, *n* (%)	11 (69)	5 (63)	6 (75)	1.000
MRA, *n* (%)	15 (94)	7 (88)	8 (100)	1.000
SGLT2i, *n* (%)	8 (50)	4 (50)	4 (50)	1.000
Furosemide, *n* (%)	3 (19)	2 (25)	1 (13)	1.000

Values are mean ± SD, median (interquartile interval), or *n* (%). *ACEi* angiotensin-converting-enzyme inhibitors, *ARB* angiotensin receptor blockers, *ARNI* angiotensin receptor–neprilysin inhibitors, *BMI* body mass index, *COPD* chronic obstructive pulmonary disease, *HFmrEF* heart failure with mildly reduced ejection fraction, *HFrEF* heart failure with reduced ejection fraction, *LVEF* left ventricular ejection fraction, *MRA* mineralocorticoid receptor antagonists, *NYHA* New York Heart Association, *SGLT2i* sodium–glucose cotransporter-2 inhibitors

At the baseline multichannel recording, in line with the use of beta-blockers, patients had mild bradycardia (mean heart rate 59 ± 11 bpm), while beat-to-beat BP values were in the normal ranges (Table 2). The mean cardio-vagal baroreflex gain was 5.6 ± 3.1 ms/mmHg, and time-domain and frequency-domain HRV parameters were significantly lower compared with reference values in the general population (Table 2).

**Table 2:** Baseline vital parameters and autonomic assessment in the study population distinguished into HFmrEF and HFrEF categories

Variables	All patients (*n* = 16)	HFmrEF (*n* = 8)	HFrEF (*n* = 8)	*p*-Value
Time domain				
Respiratory rate, breaths per minute	18 ± 4	19 ± 4	16 ± 4	0.164
Heart rate, bpm	59 ± 11	60 ± 13	59 ± 8	0.842
NN, ms	1042 ± 169	1040 ± 193	1043 ± 155	0.968
SDNN, ms	25 ± 7	25 ± 9	25 ± 6	0.947
rMSSD, ms	25 ± 20	26 ± 22	25 ± 20	0.913
Systolic BP, mmHg	106 ± 13	111 ± 11	100 ± 12	0.072
Diastolic BP, mmHg	60 ± 11	64 ± 13	57 ± 9	0.229
Mean BP, mmHg	76 ± 11	80 ± 11	73 ± 10	0.201
Systolic BP-SD, mmHg	4.9 (3.5–6.4)	5.2 (3.5–11.3)	4.7 (3.4–5.7)	0.584
Baroreflex gain, ms/mmHg	5.6 ± 3.1	5.1 ± 2.9	6.2 ± 3.4	0.497
Frequency domain				
Total power, ms^2^	763 (236–929)	817 (169–1260)	574 (254–874)	0.833
LF power, ms^2^	139 (57–232)	136 (79–193)	139 (56–241)	0.916
Ln(LF)	4.9 ± 0.8	4.8 ± 0.9	5.1 ± 0.7	0.232
LF, n.u.	58 ± 21	61 ± 22	56 ± 21	0.649
HF power, ms^2^	94 (39–182)	113 (36–182)	88 (41–221)	0.916
Ln(HF)	4.7 ± 1.3	4.6 ± 1.3	4.9 ± 1.4	0.306
HF, n.u.	42 ± 21	39 ± 21	45 ± 21	0.615
LF/HF	1.9 ± 1.3	2.0 ± 1.1	1.8 ± 1.6	0.783

Values are mean ± SD and median (interquartile interval). *BP* blood pressure, *HF* high frequency, *HFmrEF* heart failure with mildly reduced ejection fraction, *HFrEF* heart failure with reduced ejection fraction, *LF* low frequency, *NN* normal-to-normal heartbeat interval, *rMSSD* root mean square of the successive differences between normal-to-normal intervals, *SDNN* standard deviation of the normal-to-normal intervals

Except for body mass index and LVEF, no significant differences were observed comparing patients with either HFmrEF or HFrEF (Tables 1, 2).

### Acute effects of tVNS

The maximum tolerated current intensity was similar for left-sided and right-sided tVNS, with a median value of 23 (range 10–44) mA at the left tragus and 23 (range 12–33) mA at the right tragus, respectively. No patients complained of discomfort or any other adverse effects during the stimulation phases.

As detailed in Table 3, tVNS did not affect vital parameters, observing only mild and nonsignificant reductions in heart rate and increase in BP values over the protocol.

**Table 3:** Comparisons of vital parameters and autonomic measures in the study population between baseline recording and tVNS phases

Variables	Baseline	Left tVNS 23 (10–44) mA	Right tVNS 23 (12–33) mA	ANOVA main effect *p*-Value
Time domain				
Respiratory rate, breaths per minute	18 ± 4	17 ± 3	18 ± 3	0.110
Heart rate, bpm	59 ± 11	59 ± 11	58 ± 10	0.189
NN, ms	1042 ± 169	1051 ± 174	1057 ± 170	0.274
SDNN, ms	25 ± 7	31 ± 12	33 ± 17	0.127
rMSSD, ms	25 ± 20	26 ± 18	28 ± 21	0.378
Systolic BP, mmHg	106 ± 13	107 ± 11	108 ± 13	0.508
Diastolic BP, mmHg	60 ± 11	62 ± 10	61 ± 10	0.508
Mean BP, mmHg	76 ± 11	78 ± 10	77 ± 10	0.568
Systolic BP-SD, mmHg	4.9 (3.5–6.4)	4.6 (3.9–7.3)	4.7 (3.8–6.1)	0.507
Baroreflex gain, ms/mmHg	5.6 ± 3.1	6.1 ± 2.4	7.5 ± 3.8	0.003^*^
Frequency domain				
Total power, ms^2^	763 (236–929)	765 (482–1125)	717 (483–1282)	0.589
LF power, ms^2^	139 (57–232)	122 (76–178)	130 (75–229)	0.848
Ln(LF)	4.9 ± 0.8	4.9 ± 0.8	4.9 ± 0.9	0.804
LF, n.u	58 ± 21	53 ± 24	50 ± 24	0.138
HF power, ms^2^	94 (39–182)	104 (41–164)	138 (54–311)	0.571
Ln(HF)	4.5 ± 1.5	4.6 ± 1.4	4.9 ± 1.3	0.143
HF, n.u.	42 ± 21	47 ± 24	50 ± 24	0.129
LF/HF	1.9 ± 1.3	1.9 ± 1.7	1.7 ± 1.6	0.559

Values are mean ± SD and median (interquartile interval). *BP* blood pressure, *HF* high frequency, *HFmrEF* heart failure with mildly reduced ejection fraction, *HFrEF* heart failure with reduced ejection fraction, *LF* low frequency, *NN* normal-to-normal heartbeat interval, *rMSSD* root mean square of the successive differences between normal-to-normal intervals, *SDNN* standard deviation of the normal-to-normal intervals, *tVNS* transcutaneous vagus nerve stimulation. ^*^Bonferroni post hoc test pairwise comparisons *p*-values: left tVNS versus baseline *p* = 0.914; right tVNS versus baseline *p* < 0.001; and left tVNS versus right tVNS *p* = 0.163

**Fig. 1:**
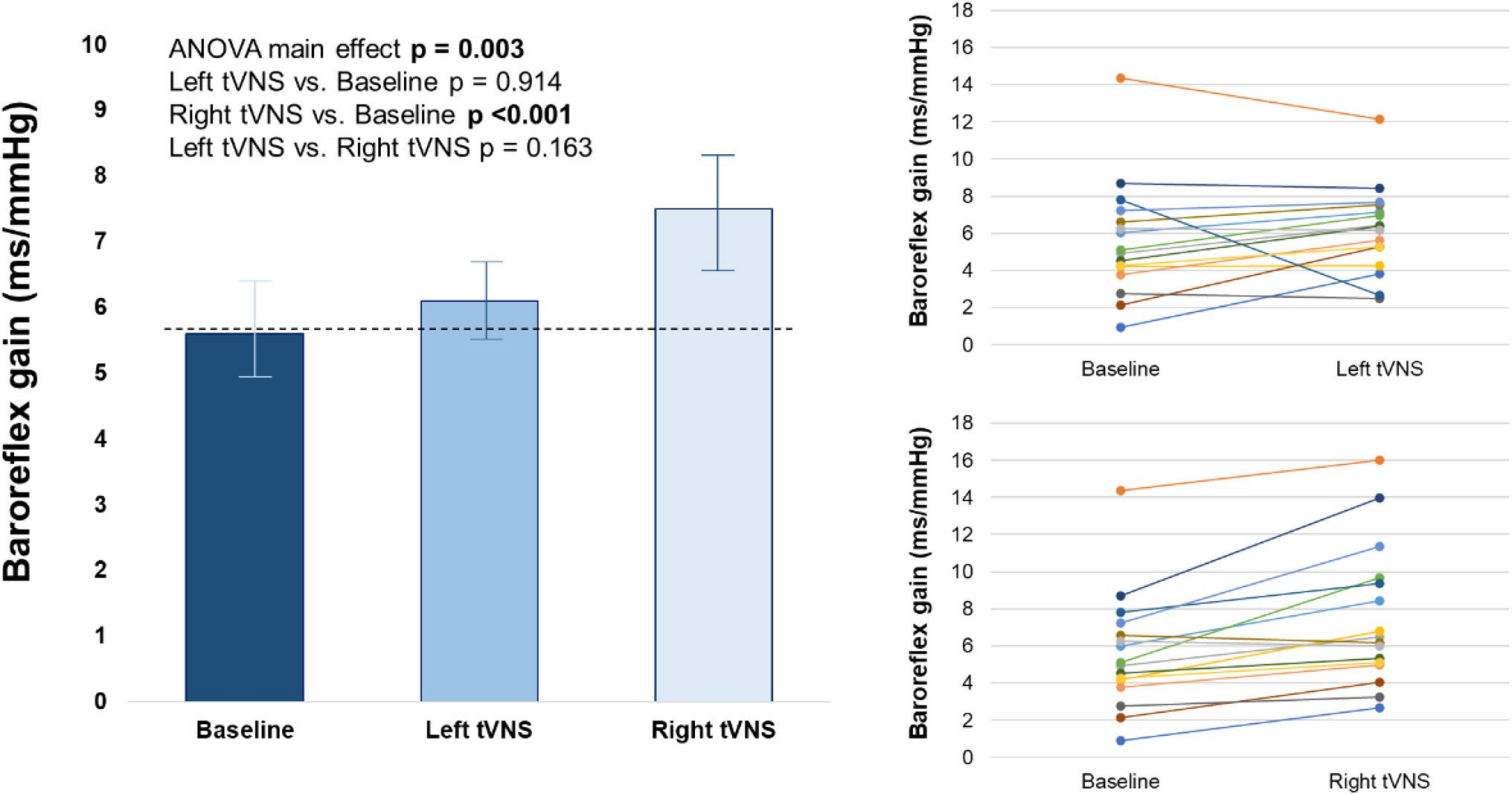
Effects of tVNS on cardio-vagal baroreflex gain in patients with chronic heart failure. Acute right-sided—but not left-sided—tVNS increased cardio-vagal baroreflex gain in the study population. Each patient underwent left-sided and right-sided tVNS during the same experimental session, in a randomized order, and separated by a 10-min recovery. One-way ANOVA for repeated measure was used, with Bonferroni correction for post hoc pairwise companions. *tVNS* transcutaneous vagus nerve stimulation

**Fig. 2:**
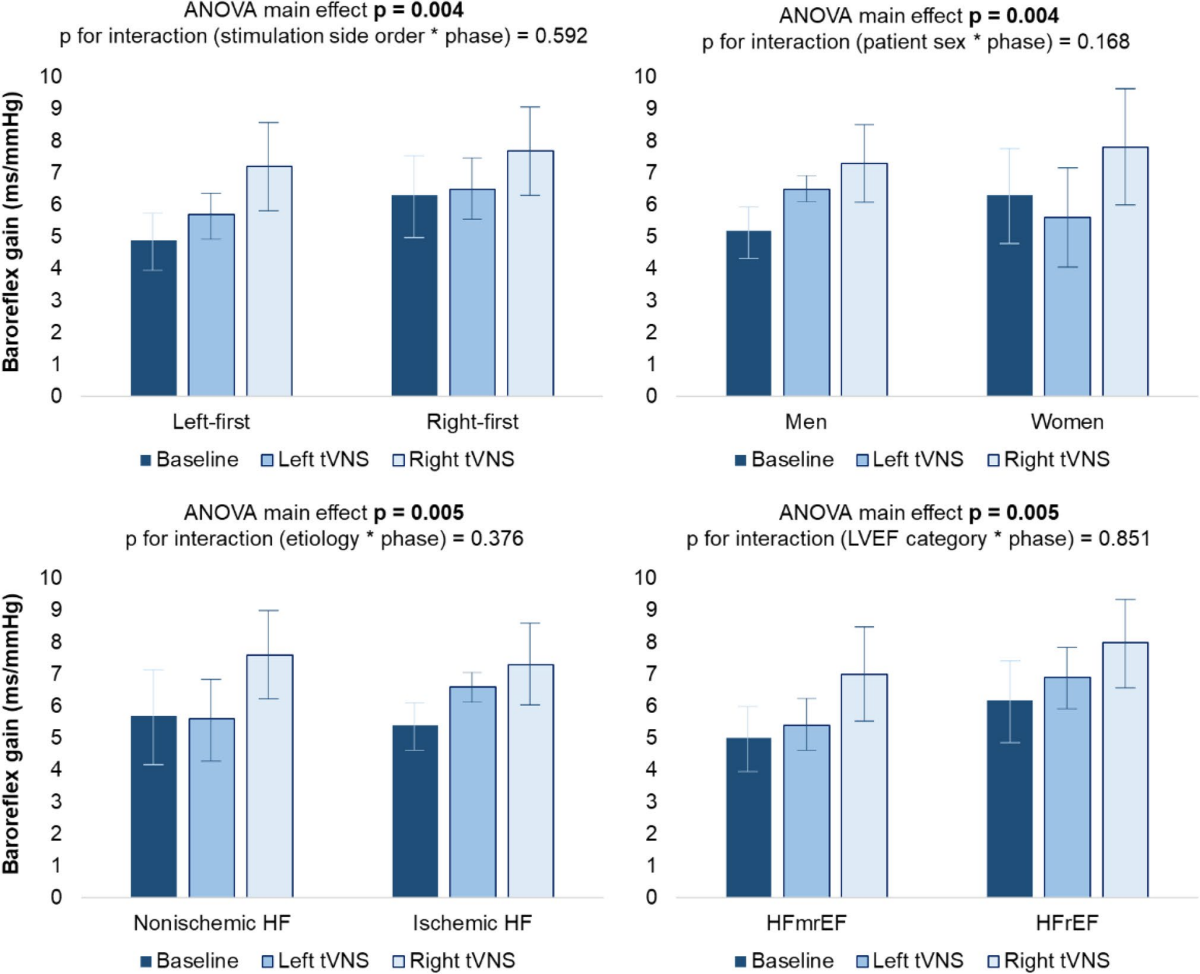
Effects of tVNS on cardio-vagal baroreflex gain across patient subgroups. The efficacy of acute right-sided tVNS in increasing cardio-vagal baroreflex gain was consistent across different subgroups in the study population. One-way ANOVA for repeated measure was used. *HFmrEF* heart failure with mildly reduced ejection fraction, *HFrEF* heart failure with reduced ejection fraction, *tVNS* transcutaneous vagus nerve stimulation

Right-sided—but not left-sided—tVNS was associated with a significant increase in cardio-vagal baroreflex gain with a ∆ of 1.9 ± 1.6 ms/mmHg from baseline (Bonferroni-corrected *p*-value < 0.001; Fig. 1). The association between right-sided tVNS and cardio-vagal baroreflex gain increase was confirmed at subgroup analysis, comparing stimulation-side order (*p*-value for interaction 0.59), patient sex (*p*-value for interaction 0.17), LVEF category (*p*-value for interaction 0.85), and CHF etiology (*p*-value for interaction 0.38; Fig. 2).

**Fig. 3:**
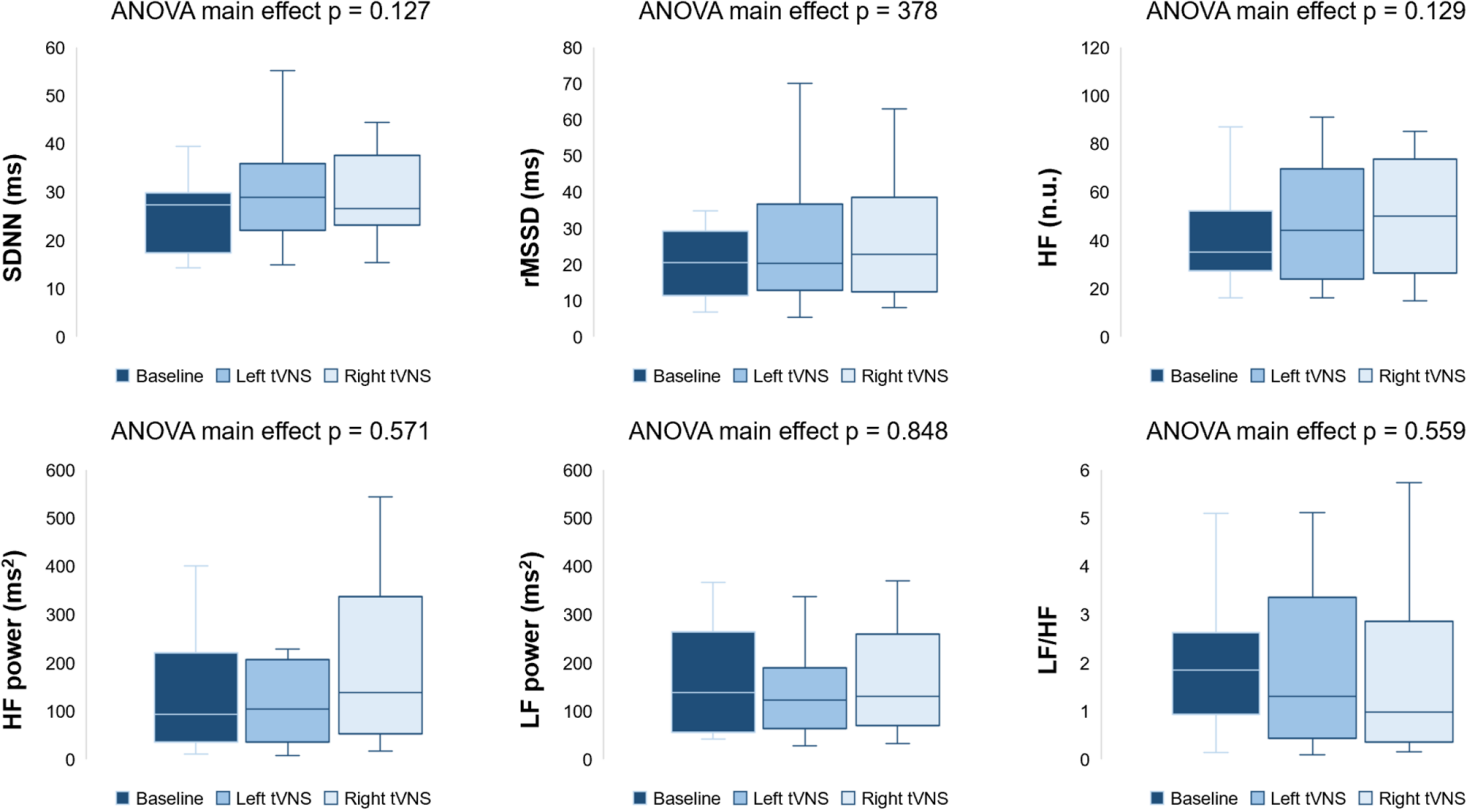
Effects of tVNS on HRV parameters in patients with chronic heart failure. Acute right-sided tVNS was associated with nonsignificant changes in time-domain and frequency-domain HRV parameters toward vagal predominance (all *p* > 0.05). One-way ANOVA for repeated measure was used. Individual data points for each parameters are reported in Supplemental Figures 1, 2, and 3. *HF* high frequency, *LF* low frequency, *rMSSD* root mean square of the successive differences between normal-to-normal intervals, *SDNN* standard deviation of the normal-to-normal intervals, *tVNS* transcutaneous vagus nerve stimulation

As for HRV, compared with the baseline, right-sided tVNS was associated with nonsignificant changes toward vagal predominance (Fig. 3; all *p* > 0.05).

## Discussion

This is the first study evaluating the acute effects of tVNS, obtained through the Parasym Device, in patients with CHF and LVEF < 50%. In this cohort, tVNS was well-tolerated, and no adverse effects were reported. Right-sided tVNS was associated with a statistically significant increase in cardio-vagal baroreflex gain, accompanied by nonsignificant changes in HRV parameters toward vagal predominance, while left-sided tVNS was not. These findings were consistent independently of stimulation side order, patient sex, CHF etiology, and LVEF category.

Despite the recent advances in drug and device treatment, patients with CHF still are at high risk of recurrent hospitalizations, malignant arrhythmias, and early mortality [26]. Since residual sympathovagal imbalance contributes to disease progression and adverse events even in patients on optimal therapy [1, 9], novel approaches for neuromodulation have been proposed not only to decrease sympathetic activity but also to increase vagal signaling [22, 30]. Despite encouraging preliminary findings, no strategy has shown satisfactory results in clinical studies such that it has been translated into routine practice so far [30]. Most notably, while invasive VNS failed to show clinical benefits in the context of randomized trials [31], optimal criteria for patient selection and the invasive nature have limited the use of BAT despite its potential efficacy. [12, 13]

To overcome these limits, tVNS has been proposed as a noninvasive alternative to improve sympathovagal balance [30]. After various preclinical studies demonstrating that tVNS may improve sympathovagal balance and prevent adverse cardiac remodeling [15, 17, 32], preliminary clinical studies have shown some benefits in the short term for patients with myocardial infarction [33], and in the medium term for those with atrial fibrillation [34], or HFpEF. [20]

To date, the autonomic effects of tVNS have been tested almost exclusively in healthy subjects, mainly evaluating HRV as the endpoint and reporting mixed findings [35]. Considering that multiple endogenous and/or exogenous factors may influence HRV (e.g., respiration, body temperature, comorbidities, etc.) [28, 36], the use of alternative markers of autonomic function has been advocated to assess tVNS efficacy. In this respect, while direct neural recording represents the gold standard method, the assessment of cardio-vagal baroreflex gain has been proposed as well [19, 21]. Indeed, among patients with CHF, reduced cardio-vagal baroreflex gain was associated with a worse clinical profile and identified as an independent predictor of mortality in multivariable regression analyses. [8, 9]

For the first time, in the present study, the tolerability and potential efficacy of acute tVNS were tested in patients with CHF and systolic dysfunction, which are characterized by a more severe sympathovagal imbalance [1, 37]. Accordingly, in our cohort, both baroreflex [38] and HRV measures [39] were significantly lower compared with those reported in the general population, despite the fact that most of the patients were receiving optimized therapies according to the latest guidelines (88% on beta-blockers, 69% on sacubitril-valsartan, 94% on mineralocorticoid-receptor antagonists, and 50% on sodium–glucose cotransporter-2 inhibitors). Of note, right-sided tVNS was associated with a mean 34% increase in cardio-vagal baroreflex gain (*p* < 0.001), while no significant changes were observed for HRV parameters. Furthermore, tVNS was not associated with any adverse effects or discomfort for the patients.

Though the precise link between tVNS and baroreflex is unclear, similar findings had been previously reported in healthy subjects [19, 21]. In the study by Antonino et al., 15-min active tVNS, but not sham stimulation, was associated with a mean 24% increase in cardio-vagal baroreflex gain compared with baseline values in 13 volunteers [21]. Similar findings were reported by Bretherton et al. in 69 individuals aged ≥ 55 years free of cardiovascular disease, in which tVNS was associated with a mean 22% increase in cardio-vagal baroreflex gain. [19]

The mechanisms behind this relation, as well as the potential clinical implications, are not completely understood [40]. By using functional magnetic resonance during tVNS, a consistent activation of the nucleus tractus solitarius has been documented [41]. Since the nucleus tractus solitarius represents the main relay station for arterial baroreflex afferents [42], a central interaction between tVNS and baroreflex gain has been hypothesized. In this respect, also the direct stimulation of afferent vagal fibers has been associated with improved baroreflex function in an experimental rat model of myocardial infarction [43]. However, since baroreceptors also modulate sympathetic efferences, cardio-vagal baroreflex gain cannot be considered independent by other hemodynamic and autonomic influences. Therefore, to gain a deeper understanding of the autonomic effects of tVNS, future studies evaluating both the cardio-vagal and vascular-sympathetic limbs of baroreflex are warranted.

While the used stimulation side had not been reported in the previous studies [19, 21], only right-sided tVNS was associated with improved cardio-vagal baroreflex gain in this work. While concerns about the safety of right-sided stimulation had been hypothesized for invasive VNS [24, 25], due to the potential risk of a stronger sinus node inhibition [24, 25], right and left vagus stimulation were associated with similar changes in heart rate in the CHF patients enrolled in the ANTHEM-HF trial [44]. Furthermore, while left-sided stimulation has been arbitrarily chosen in most of the clinical studies conducted so far, no adverse effects have been reported with either right-sided or left-sided tVNS [23]. Interestingly, some functional asymmetry has been reported for baroreflex function. Indeed, right carotid baroreflex activation was more effective than left stimulation in modulating HRV [45, 46], and a role of the ipsilateral central projections to the nucleus tractus solitarius was hypothesized. While the clinical efficacy of right versus left BAT has not been compared in CHF so far, right-sided BAT (*n* = 127) was more effective than left-sided BAT (*n* = 88) in lowering BP among 215 patients with resistant hypertension. [47]

Whether the stimulation side may affect the efficacy of tVNS on other endpoints beyond cardio-vagal baroreflex gain remains to be investigated. Nevertheless, taken together, these findings underscore the importance of comparing and reporting the stimulation side when testing novel neuromodulation strategies.

### Study limitations

The small sample size may not allow for the immediate translation of the findings to other cohorts, considering the heterogeneity characterizing the CHF population. Nonetheless, the inclusion of patients on stable optimized medical therapy followed-up at the outpatient clinic of our tertiary center, the study cohort may constitute a snapshot of modern well-treated real-life CHF patients. In the absence of definitive evidence about the interactions with the Parasym Device, only patients with no implantable cardiac devices were enrolled. Though no safety issues were reported in patients with pacemakers [20], dedicated studies should assess the safety of tVNS in this subset. In the present study, no “sham stimulation” protocol was performed: while the optimal option for a reliable “sham stimulation” is an object of debate due to the observation that lobe stimulation may itself activate central autonomic areas [23, 48], performing both left-sided and right-sided tVNS in a randomized order constituted a fair “control condition,” beyond providing an answer to an open research question [23]. In this respect, confirming that only right-sided tVNS was effective in increasing cardio-vagal baroreflex gain independently of stimulation side order ruled out concerns about the carry-over effect related to the initial stimulation side over the protocol.

The optimal parameters for tVNS are unclear and may vary according to the device used and the study endpoint [23]. In patients with postural tachycardia syndrome, a stimulation frequency of 25 Hz was identified as the optimal one to increase the HF component of HRV [49]. Therefore, we could not exclude that having used a stimulation frequency of 30 Hz may have affected the findings of the current study. Considering the differences in the study populations (e.g., age, sex, severity of autonomic imbalance, and medications), further studies should investigate the optimal stimulation parameters in patients with CHF. [23]

While spontaneous cardio-vagal baroreflex gain is an accurate, reproducible, and widely available noninvasive parameter [29], which has been shown to retain a strong prognostic value in CHF [9], the clinical significance of its modulation remains to be confirmed. Indeed, the reduction in cardio-vagal baroreflex gain observed in CHF patients may not exclusively indicate a pathophysiological process. As evidenced in healthy sedentary adults [50], lower cardio-vagal baroreflex gain can result from arterial stiffening, a natural aspect of aging, particularly in sedentary individuals, rather than serving as a direct marker of disease [51]. However, a significant improvement in cardio-vagal baroreflex gain through physical training in patients with CHF has been associated with reduced cardiac mortality during follow-up [52]. While the contribution of other beneficial mechanisms secondary to physical training cannot be excluded, these findings underscore the potential importance of enhancing cardio-vagal baroreflex gain in CHF management. Notwithstanding, other studies are expected to assess the autonomic effects of tVNS in CHF patients by using direct measures of sympathetic and vagus nerve activity, assessed through microneurography.

The use of anti-neurohormonal drugs can impact both cardiovascular parameters (such as BP and heart rate) and autonomic functions (including cardio-vagal baroreflex gain). However, due to safety concerns, these therapies were not discontinued in study patients. While this may have influenced the effects of tVNS on these parameters, it is valuable to provide data on CHF patients treated in accordance with the latest guidelines. This approach reflects the potential clinical application of tVNS as an adjunct therapeutic strategy.

Finally, in this study, only the acute effects of tVNS were evaluated, which are unlikely to provide clinical benefits. However, together with the promising findings derived from preclinical models [15, 32], these results encourage designing further studies to evaluate the efficacy of chronic tVNS.

## Conclusions

Acute right-sided tVNS is safe and well tolerated and improves cardio-vagal baroreflex gain in adults with systolic CHF. Considering the pathophysiological and prognostic significance of reduced baroreflex function in this population, right-sided tVNS may prove valuable as a novel noninvasive and cost-effective strategy for neuromodulation.

Future studies should now test the safety and effectiveness of chronic tVNS in patients with systolic CHF, evaluating the potential benefits on autonomic balance, neurohormonal activation, cardiac function, and other clinically relevant endpoints.

## Supplementary Information

Below is the link to the electronic supplementary material.

Supplementary file1 (DOCX 354 KB)

## Data Availability

Upon reasonable request.

## Abbreviations

BMI: Body mass index
BP: Blood pressure
CHF: Chronic heart failure
HF: High frequency
HFmrEF: Heart failure with mildly reduced ejection fraction
HFpEF: Heart failure with preserved ejection fraction
HFrEF: Heart failure with reduced ejection fraction
HRV: Heart rate variability
LF: Low frequency
LVEF: Left ventricular ejection fraction
RMSSD: Root mean square of successive differences
SD: Standard deviation
SDNN: Standard deviation of all normal-to-normal intervals
tVNS: Transcutaneous vagus nerve stimulation

## References

[1] Floras JS, Ponikowski P (2015) The sympathetic/parasympathetic imbalance in heart failure with reduced ejection fraction. Eur Heart J 36:1974–1982b 25975657 10.1093/eurheartj/ehv087 PMC4528097

[2] Barretto ACP, Santos AC, Munhoz R, Rondon MUPB, Franco FG, Trombetta IC, Roveda F, de Matos LNJ, Braga AMW, Middlekauff HR, Negrão CE (2009) Increased muscle sympathetic nerve activity predicts mortality in heart failure patients. Int J Cardiol 135:302–307 18582965 10.1016/j.ijcard.2008.03.056

[3] Cohn JN, Levine TB, Olivari MT, Garberg V, Lura D, Francis GS, Simon AB, Rector T (1984) Plasma norepinephrine as a guide to prognosis in patients with chronic congestive heart failure. N Engl J Med 311:819–823 6382011 10.1056/NEJM198409273111303

[4] Meredith IT, Broughton A, Jennings GL, Esler MD (1991) Evidence of a selective increase in cardiac sympathetic activity in patients with sustained ventricular arrhythmias. N Engl J Med 325:618–624 1861695 10.1056/NEJM199108293250905

[5] Verberne HJ, Brewster LM, Somsen GA, van Eck-Smit BLF (2008) Prognostic value of myocardial 123I-metaiodobenzylguanidine (MIBG) parameters in patients with heart failure: a systematic review. Eur Heart J 29:1147–1159 18349024 10.1093/eurheartj/ehn113

[6] La Rovere MT, Pinna GD, Maestri R, Mortara A, Capomolla S, Febo O, Ferrari R, Franchini M, Gnemmi M, Opasich C, Riccardi PG, Traversi E, Cobelli F (2003) Short-term heart rate variability strongly predicts sudden cardiac death in chronic heart failure patients. Circulation 107:565–570 12566367 10.1161/01.cir.0000047275.25795.17

[7] La Rovere MT, Bigger JT, Marcus FI, Mortara A, Schwartz PJ (1998) Baroreflex sensitivity and heart-rate variability in prediction of total cardiac mortality after myocardial infarction. ATRAMI (Autonomic Tone and Reflexes After Myocardial Infarction) investigators. Lancet 351:478–484 9482439 10.1016/s0140-6736(97)11144-8

[8] La Rovere MT, Pinna GD, Maestri R, Robbi E, Caporotondi A, Guazzotti G, Sleight P, Febo O (2009) Prognostic implications of baroreflex sensitivity in heart failure patients in the beta-blocking era. J Am Coll Cardiol 53:193–199 19130988 10.1016/j.jacc.2008.09.034

[9] Giannoni A, Gentile F, Buoncristiani F, Borrelli C, Sciarrone P, Spiesshoefer J, Bramanti F, Iudice G, Javaheri S, Emdin M, Passino C (2022) Chemoreflex and baroreflex sensitivity hold a strong prognostic value in chronic heart failure. JACC Heart Fail 10:662–676 36049816 10.1016/j.jchf.2022.02.006

[10] Chua TP, Ponikowski P, Webb-Peploe K, Harrington D, Anker SD, Piepoli M, Coats AJ (1997) Clinical characteristics of chronic heart failure patients with an augmented peripheral chemoreflex. Eur Heart J 18:480–486 9076386 10.1093/oxfordjournals.eurheartj.a015269

[11] Aimo A, Saccaro LF, Borrelli C (2021) The ergoreflex: how the skeletal muscle modulates ventilation and cardiovascular function in health and disease. Eur J Heart Fail 23:1458–1467 34268843 10.1002/ejhf.2298 PMC9292527

[12] Coats AJS, Abraham WT, Zile MR, Lindenfeld JA, Weaver FA, Fudim M, Bauersachs J, Duval S, Galle E, Zannad F (2022) Baroreflex activation therapy with the Barostim™ device in patients with heart failure with reduced ejection fraction: a patient level meta-analysis of randomized controlled trials. Eur J Heart Fail 24:1665–1673 35713888 10.1002/ejhf.2573 PMC9796660

[13] Gentile F, Passino C, Emdin M, Giannoni A (2022) Baroreflex activation therapy in heart failure: targeting the right patient. Eur J Heart Fail 24:1674–1676 35851979 10.1002/ejhf.2627

[14] Giannoni A, Gentile F, Passino C (2022) Bioelectronic medicine and its applications in cardiology. Eur Heart J 43:4453–4455 35751532 10.1093/eurheartj/ehac343

[15] Yu L, Wang S, Zhou X, Wang Z, Huang B, Liao K, Saren G, Chen M, Po SS, Jiang H (2016) Chronic intermittent low-level stimulation of tragus reduces cardiac autonomic remodeling and ventricular arrhythmia inducibility in a post-infarction canine model. JACC Clin Electrophysiol 2:330–339 29766893 10.1016/j.jacep.2015.11.006

[16] Chen M, Chen H, Wang Z, Pan Y, Hu H, Wang S, Yuan Y, Wang Z, Jiang H (2022) Non-invasive tragus stimulation improves cardiac post-ischemic remodeling by regulating cardiac parasympathetic activity. ESC Heart Fail 9:4129–4138 36085552 10.1002/ehf2.14146 PMC9773748

[17] Elkholey K, Niewiadomska M, Morris L, Whyte S, Houser J, Humphrey MB, Stavrakis S (2022) Transcutaneous vagus nerve stimulation ameliorates the phenotype of heart failure with preserved ejection fraction through its anti-inflammatory effects. Circ Heart Fail 15:e009288 35862007 10.1161/CIRCHEARTFAILURE.122.009288 PMC9388556

[18] Clancy JA, Mary DA, Witte KK, Greenwood JP, Deuchars SA, Deuchars J (2014) Non-invasive vagus nerve stimulation in healthy humans reduces sympathetic nerve activity. Brain Stimul 7:871–877 25164906 10.1016/j.brs.2014.07.031

[19] Bretherton B, Atkinson L, Murray A, Clancy J, Deuchars S, Deuchars J (2019) Effects of transcutaneous vagus nerve stimulation in individuals aged 55 years or above: potential benefits of daily stimulation. Aging (Albany NY) 11:4836–4857 31358702 10.18632/aging.102074 PMC6682519

[20] Stavrakis S, Elkholey K, Morris L, Niewiadomska M, Asad ZUA, Humphrey MB (2022) Neuromodulation of inflammation to treat heart failure with preserved ejection fraction: a pilot randomized clinical trial. J Am Heart Assoc 11:e023582 35023349 10.1161/JAHA.121.023582 PMC9238491

[21] Antonino D, Teixeira AL, Maia-Lopes PM, Souza MC, Sabino-Carvalho JL, Murray AR, Deuchars J, Vianna LC (2017) Non-invasive vagus nerve stimulation acutely improves spontaneous cardiac baroreflex sensitivity in healthy young men: a randomized placebo-controlled trial. Brain Stimul 10:875–881 28566194 10.1016/j.brs.2017.05.006

[22] van Bilsen M, Patel HC, Bauersachs J, Böhm M, Borggrefe M, Brutsaert D, Coats AJS, de Boer RA, de Keulenaer GW, Filippatos GS, Floras J, Grassi G, Jankowska EA, Kornet L, Lunde IG, Maack C, Mahfoud F, Pollesello P, Ponikowski P, Ruschitzka F, Sabbah HN, Schultz HD, Seferovic P, Slart RHJA, Taggart P, Tocchetti CG, Van Laake LW, Zannad F, Heymans S, Lyon AR (2017) The autonomic nervous system as a therapeutic target in heart failure: a scientific position statement from the Translational Research Committee of the Heart Failure Association of the European Society of Cardiology. Eur J Heart Fail 19:1361–1378 28949064 10.1002/ejhf.921

[23] Farmer AD, Strzelczyk A, Finisguerra A, Gourine AV, Gharabaghi A, Hasan A, Burger AM, Jaramillo AM, Mertens A, Majid A, Verkuil B, Badran BW, Ventura-Bort C, Gaul C, Beste C, Warren CM, Quintana DS, Hämmerer D, Freri E, Frangos E, Tobaldini E, Kaniusas E, Rosenow F, Capone F, Panetsos F, Ackland GL, Kaithwas G, O’Leary GH, Genheimer H, Jacobs HIL, Van Diest I, Schoenen J, Redgrave J, Fang J, Deuchars J, Széles JC, Thayer JF, More K, Vonck K, Steenbergen L, Vianna LC, McTeague LM, Ludwig M, Veldhuizen MG, De Couck M, Casazza M, Keute M, Bikson M, Andreatta M, D’Agostini M, Weymar M, Betts M, Prigge M, Kaess M, Roden M, Thai M, Schuster NM, Montano N, Hansen N, Kroemer NB, Rong P, Fischer R, Howland RH, Sclocco R, Sellaro R, Garcia RG, Bauer S, Gancheva S, Stavrakis S, Kampusch S, Deuchars SA, Wehner S, Laborde S, Usichenko T, Polak T, Zaehle T, Borges U, Teckentrup V, Jandackova VK, Napadow V, Koenig J (2020) International consensus based review and recommendations for minimum reporting standards in research on transcutaneous vagus nerve stimulation (Version 2020). Front Hum Neurosci 14:568051 33854421 10.3389/fnhum.2020.568051 PMC8040977

[24] Ng GA, Brack KE, Coote JH (2001) Effects of direct sympathetic and vagus nerve stimulation on the physiology of the whole heart—a novel model of isolated Langendorff perfused rabbit heart with intact dual autonomic innervation. Exp Physiol 86:319–329 11471534 10.1113/eph8602146

[25] Brack KE, Coote JH, Ng GA (2004) Interaction between direct sympathetic and vagus nerve stimulation on heart rate in the isolated rabbit heart. Exp Physiol 89:128–139 15109218 10.1113/expphysiol.2003.002654

[26] McDonagh TA, Metra M, Adamo M, Gardner RS, Baumbach A, Böhm M, Burri H, Butler J, Čelutkienė J, Chioncel O, Cleland JGF, Coats AJS, Crespo-Leiro MG, Farmakis D, Gilard M, Heymans S, Hoes AW, Jaarsma T, Jankowska EA, Lainscak M, Lam CSP, Lyon AR, McMurray JJV, Mebazaa A, Mindham R, Muneretto C, Francesco Piepoli M, Price S, Rosano GMC, Ruschitzka F, Kathrine Skibelund A, ESC Scientific Document Group (2021) ESC Guidelines for the diagnosis and treatment of acute and chronic heart failure. Eur Heart J 2021(42):3599–3726

[27] Lang RM, Badano LP, Mor-Avi V, Afilalo J, Armstrong A, Ernande L, Flachskampf FA, Foster E, Goldstein SA, Kuznetsova T, Lancellotti P, Muraru D, Picard MH, Rietzschel ER, Rudski L, Spencer KT, Tsang W, Voigt J-U (2015) Recommendations for cardiac chamber quantification by echocardiography in adults: an update from the American Society of Echocardiography and the European Association of Cardiovascular Imaging. J Am Soc Echocardiogr 28:1-39.e14 25559473 10.1016/j.echo.2014.10.003

[28] Heart rate variability: standards of measurement, physiological interpretation and clinical use. Task Force of the European Society of Cardiology and the North American Society of Pacing and Electrophysiology. *Circulation* 1996;**93**:1043–1065. 8598068

[29] Bernardi L, De Barbieri G, Rosengård-Bärlund M, Mäkinen V-P, Porta C, Groop P-H (2010) New method to measure and improve consistency of baroreflex sensitivity values. Clin Auton Res 20:353–361 20700641 10.1007/s10286-010-0079-1

[30] Fudim M, Abraham WT, von Bardeleben RS, Lindenfeld J, Ponikowski PP, Salah HM, Khan MS, Sievert H, Stone GW, Anker SD, Butler J (2021) Device therapy in chronic heart failure: JACC state-of-the-art review. J Am Coll Cardiol 78:931–956 34446165 10.1016/j.jacc.2021.06.040 PMC9941752

[31] Dusi V, Ferrari GMD (2021) Vagal stimulation in heart failure. Herz 46:541 34716778 10.1007/s00059-021-05076-5 PMC8642334

[32] Wang Z, Yu L, Wang S, Huang B, Liao K, Saren G, Tan T, Jiang H (2014) Chronic intermittent low-level transcutaneous electrical stimulation of auricular branch of vagus nerve improves left ventricular remodeling in conscious dogs with healed myocardial infarction. Circ Heart Fail 7:1014–1021 25332149 10.1161/CIRCHEARTFAILURE.114.001564

[33] Yu L, Huang B, Po SS, Tan T, Wang M, Zhou L, Meng G, Yuan S, Zhou X, Li X, Wang Z, Wang S, Jiang H (2017) Low-level tragus stimulation for the treatment of ischemia and reperfusion injury in patients with ST-segment elevation myocardial infarction: a proof-of-concept study. JACC Cardiovasc Interv 10:1511–1520 28797427 10.1016/j.jcin.2017.04.036

[34] Stavrakis S, Stoner JA, Humphrey MB, Morris L, Filiberti A, Reynolds JC, Elkholey K, Javed I, Twidale N, Riha P, Varahan S, Scherlag BJ, Jackman WM, Dasari TW, Po SS (2020) TREAT AF (Transcutaneous Electrical Vagus Nerve Stimulation to Suppress Atrial Fibrillation): a randomized clinical trial. JACC Clin Electrophysiol 6:282–291 32192678 10.1016/j.jacep.2019.11.008 PMC7100921

[35] Wolf V, Kühnel A, Teckentrup V, Koenig J, Kroemer NB (2021) Does transcutaneous auricular vagus nerve stimulation affect vagally mediated heart rate variability? A living and interactive Bayesian meta-analysis. Psychophysiology 58:e13933 34473846 10.1111/psyp.13933

[36] Žunkovič B, Kejžar N, Bajrović FF (2023) Standard heart rate variability parameters-their within-session stability, reliability, and sample size required to detect the minimal clinically important effect. J Clin Med 12:3118 37176559 10.3390/jcm12093118 PMC10179119

[37] Castiglione V, Gentile F, Ghionzoli N, Chiriacò M, Panichella G, Aimo A, Vergaro G, Giannoni A, Passino C, Emdin M (2023) Pathophysiological rationale and clinical evidence for neurohormonal modulation in heart failure with preserved ejection fraction. Card Fail Rev 9:e09 37427009 10.15420/cfr.2022.23 PMC10326668

[38] Rovere MTL, Pinna GD, Raczak G (2008) Baroreflex sensitivity: measurement and clinical implications. Ann Noninvasive Electrocardiol 13(2):191–207 18426445 10.1111/j.1542-474X.2008.00219.x PMC6931942

[39] Nunan D, Sandercock GRH, Brodie DA (2010) A quantitative systematic review of normal values for short-term heart rate variability in healthy adults. Pacing Clin Electrophysiol 33:1407–1417 20663071 10.1111/j.1540-8159.2010.02841.x

[40] Butt MF, Albusoda A, Farmer AD, Aziz Q (2020) The anatomical basis for transcutaneous auricular vagus nerve stimulation. J Anat 236:588–611 31742681 10.1111/joa.13122 PMC7083568

[41] Frangos E, Ellrich J, Komisaruk BR (2015) Non-invasive access to the vagus nerve central projections via electrical stimulation of the external ear: fMRI evidence in humans. Brain Stimul 8:624–636 25573069 10.1016/j.brs.2014.11.018 PMC4458242

[42] Gerlach DA, Manuel J, Hoff A, Kronsbein H, Hoffmann F, Heusser K, Ehmke H, Diedrich A, Jordan J, Tank J, Beissner F (2019) Novel approach to elucidate human baroreflex regulation at the brainstem level: pharmacological testing during fMRI. Front Neurosci 13:193 30890917 10.3389/fnins.2019.00193 PMC6411827

[43] Kawada T, Li M, Zheng C, Shimizu S, Uemura K, Turner MJ, Yamamoto H, Sugimachi M (1985) Chronic vagal nerve stimulation improves baroreflex neural arc function in heart failure rats. J Appl Physiol 2014(116):1308–1314 10.1152/japplphysiol.00140.2014 24674859

[44] Premchand RK, Sharma K, Mittal S, Monteiro R, Dixit S, Libbus I, DiCarlo LA, Ardell JL, Rector TS, Amurthur B, KenKnight BH, Anand IS (2014) Autonomic regulation therapy via left or right cervical vagus nerve stimulation in patients with chronic heart failure: results of the ANTHEM-HF trial. J Card Fail 20:808–816 25187002 10.1016/j.cardfail.2014.08.009

[45] Furlan R, Diedrich A, Rimoldi A, Palazzolo L, Porta C, Diedrich L, Harris PA, Sleight P, Biagioni I, Robertson D, Bernardi L (2003) Effects of unilateral and bilateral carotid baroreflex stimulation on cardiac and neural sympathetic discharge oscillatory patterns. Circulation 108:717–723 12900347 10.1161/01.CIR.0000084540.91605.0C

[46] Tafil-Klawe M, Raschke F, Hildebrandt G (1990) Functional asymmetry in carotid sinus cardiac reflexes in humans. Eur J Appl Physiol Occup Physiol 60:402–405 2369914 10.1007/BF00713507

[47] de Leeuw PW, Alnima T, Lovett E, Sica D, Bisognano J, Haller H, Kroon AA (2015) Bilateral or unilateral stimulation for baroreflex activation therapy. Hypertension 65:187–192 25331845 10.1161/HYPERTENSIONAHA.114.04492

[48] Rangon C-M (2018) Reconsidering sham in transcutaneous vagus nerve stimulation studies. Clin Neurophysiol 129:2501–2502 30268709 10.1016/j.clinph.2018.08.027

[49] Diedrich A, Urechie V, Shiffer D, Rigo S, Minonzio M, Cairo B, Smith EC, Okamoto LE, Barbic F, Bisoglio A, Porta A, Biaggioni I, Furlan R (2021) Transdermal auricular vagus stimulation for the treatment of postural tachycardia syndrome. Auton Neurosci 236:102886 34634682 10.1016/j.autneu.2021.102886 PMC8939715

[50] Monahan KD, Dinenno FA, Tanaka H, Clevenger CM, DeSouza CA, Seals DR (2000) Regular aerobic exercise modulates age-associated declines in cardiovagal baroreflex sensitivity in healthy men. J Physiol 529(Pt 1):263–271 11080267 10.1111/j.1469-7793.2000.00263.x PMC2270167

[51] Monahan KD (2007) Effect of aging on baroreflex function in humans. Am J Physiol Regul Integr Comp Physiol 293:R3–R12 17442786 10.1152/ajpregu.00031.2007

[52] La Rovere MT, Bersano C, Gnemmi M, Specchia G, Schwartz PJ (2002) Exercise-induced increase in baroreflex sensitivity predicts improved prognosis after myocardial infarction. Circulation 106:945–949 12186798 10.1161/01.cir.0000027565.12764.e1

