# Supplementary material for: Acute right-sided transcutaneous vagus nerve stimulation improves cardio-vagal baroreflex gain in patients with chronic heart failure
Source: Clin Auton Res. 2024 Oct 14;35(1):75–85. doi: 10.1007/s10286-024-01074-9 (PMC11937132; doi:10.1007/s10286-024-01074-9)
Supplement: Supplementary file 1 — Supplementary file1 (DOCX 354 KB) [file 10286_2024_1074_MOESM1_ESM.docx]

**Supplemental Figure 1.** Individual data points for SDNN (standard deviation of the normal-to-normal intervals) and rMSSD (root mean square of the successive differences between normal-to-normal intervals) over the study protocol. tVNS: transcutaneous vagus nerve stimulation.


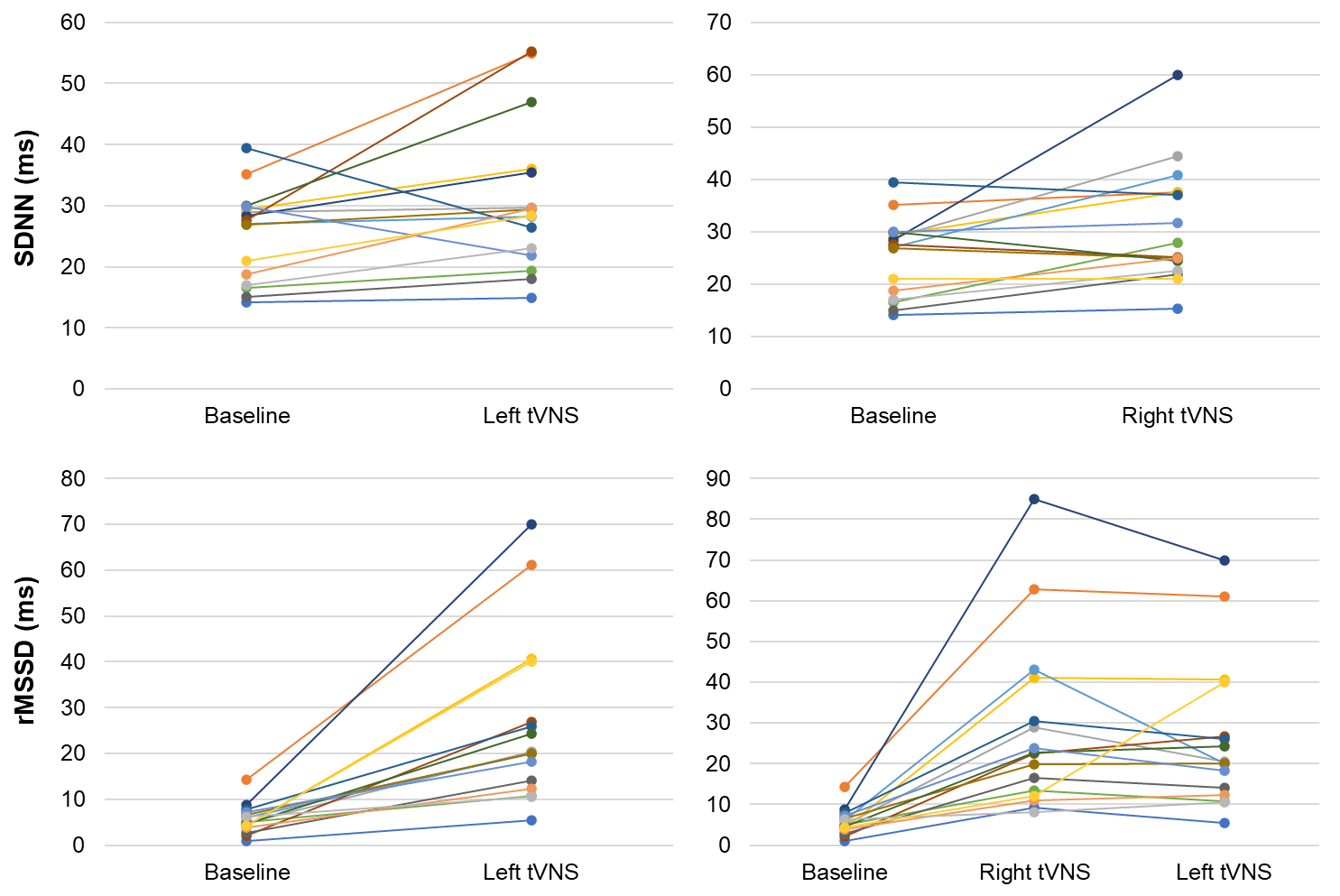


**Supplemental Figure 2.** Individual data points for high frequency (HF) components of heart rate variability expressed as either normalized units (n.u.) or global power (ms^2^) over the study protocol. tVNS: transcutaneous vagus nerve stimulation.

**
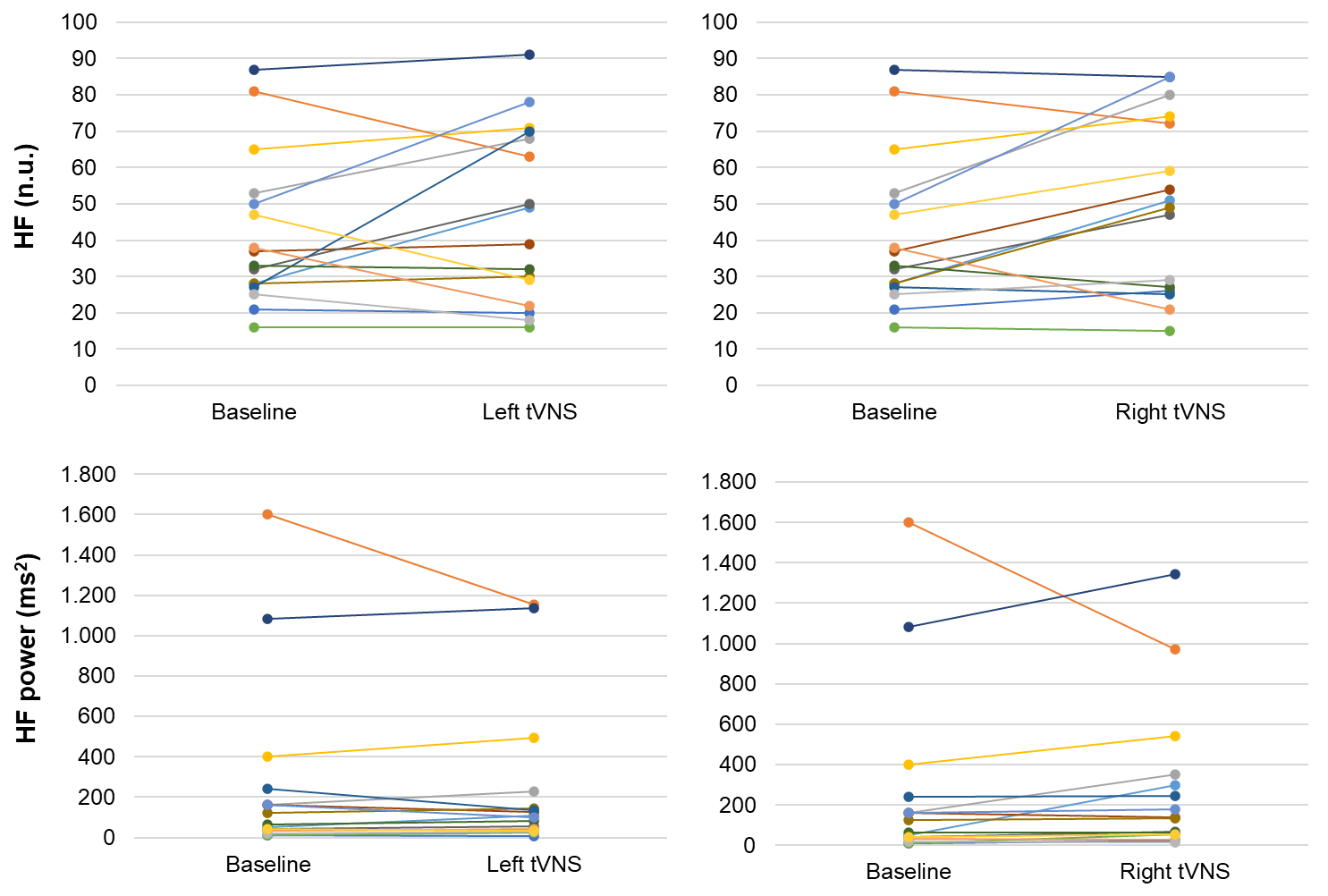
**

**Supplemental Figure 3.** Individual data points for low frequency (LF) components and LF/HF ratio over the study protocol. tVNS: transcutaneous vagus nerve stimulation.

**
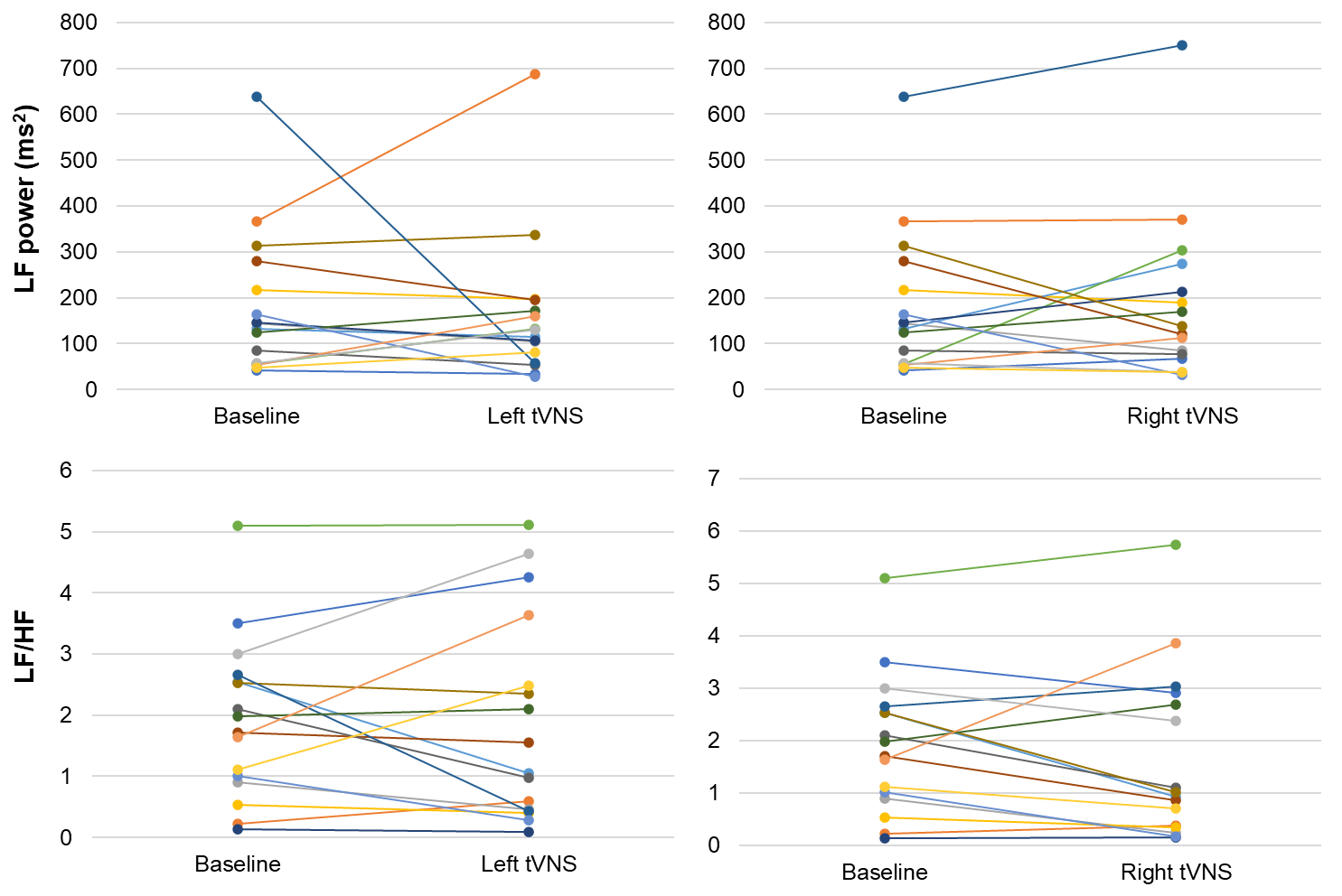
**
